# Mesenchymal Stem Cell for Prevention and Management of Intervertebral Disc Degeneration

**DOI:** 10.1155/2012/921053

**Published:** 2012-03-04

**Authors:** Umile Giuseppe Longo, Nicola Papapietro, Stefano Petrillo, Edoardo Franceschetti, Nicola Maffulli, Vincenzo Denaro

**Affiliations:** ^1^Department of Orthopaedic and Trauma Surgery, Campus Bio-Medico University, Via Alvaro del Portillo 200, Trigoria, 00128 Rome, Italy; ^2^Integrated Research Centre, Campus Bio-Medico University, Via Alvaro del Portillo 21, 00128 Rome, Italy; ^3^Centre for Sports and Exercise Medicine, Mile End Hospital, Barts and The London School of Medicine and Dentistry, 275 Bancroft Road, London E1 4DG, UK

## Abstract

Intervertebral disc degeneration (IVD) is a frequent pathological condition. Conservative management often fails, and patients with IVD degeneration may require surgical intervention. Several treatment strategies have been proposed, although only surgical discectomy and arthrodesis have been proved to be predictably effective. The aim of biological strategies is to prevent and manage IVD degeneration, improve the function, the anabolic and reparative capabilities of the nucleus pulposus and annulus fibrosus cells, and inhibit matrix degradation. At present, clinical applications are still in their infancy. Further studies are required to clarify the role of mesenchymal stem cells and gene therapy for the prevention and treatment of IVD degeneration.

## 1. Introduction

The normal intervertebral disc (IVD) has a rich extracellular matrix (ECM), and three parts are anatomically distinguished: the central gelatinous nucleus pulposus (NP), which contains chondrocyte-like cells surrounded by a more fibrous annulus fibrosus (AF) containing fibroblast-like cells along with cartilaginous endplates (EP) connecting the adjacent vertebral bodies [1–6].

The IVD matrix consists of a well-organized framework of macromolecules which are able to attract and retain water [7]. The structural components of the IVD matrix are predominantly represented by collagen and proteoglycans [8]. Collagenous proteins are mostly present in the AF, and proteoglycans are the major components of the NP. In fact, collagenous proteins comprise 70% of the outer annulus dry weight, but only 20% are located in the central NP, while proteoglycans make up 50% of the NP [8].

Functionally, the collagen provides shape and tensile strength, while proteoglycans confer tissue viscoelasticity, stiffness, and resistance to compression, through their interaction with water.

A proper balance between matrix synthesis/apposition and degradation is responsible for the maintenance of the integrity of the IVD and hence the mechanical behaviour of the IVD itself. This is related to the activity of cytokines, growth factors, enzymes, and enzyme inhibitors that act in a paracrine or/and autocrine way [9–11].

With aging, morphological and molecular changes in the IVD can induce progressive degeneration and pathologic alteration of this particular tissue [12]. The morphological changes include dehydration and tears of the AF, NP, and EP [13]. The molecular changes consist of decreased cell viability and proteoglycans synthesis and reduced diffusion of nutrient and waste products. Accumulation of apoptosis debris, degraded matrix macromolecules, and an increased degrading enzymatic activity along with a modification of the collagen distribution also represent the degenerative changes that occur at a molecular level [8, 11, 14–16]. 

A variety of growth factors have anabolic function in IVD cell metabolism, resulting in accumulation and synthesis of matrix, while the opposite effect has been observed for cytokines, since they inhibit the synthesis of IVD matrix and promote the catabolic breakdown of its components [11, 17].

Although inflammatory mediators have been detected in IVD degeneration conditions, the actual pathologic role of these mediators is either unknown or not well defined. Nitric oxide (NO), interleukin-6 (IL-6), prostaglandin E2 (PGE2), TNF-alpha, fibronectin, and matrix metalloproteinases (MMPs) are few of the many identified mediators [12, 13, 18, 19].

IL-6, NO, and PGE2 seem to exert an inhibitory effect on proteoglycan synthesis. These factors are activated by interleukin-1 (IL-1), which also plays a role in the direct degradation of the proteoglycan matrix. This direct breakdown process by IL-1 occurs through a family of enzymatic mediators known as MMPs. In the cascade of inflammatory mediators, IL-1 likely plays a major role, although the nature of such role has not been defined yet.

To find new efficient treatment options, further investigations are required to identify the mediators that promote IVD degradation or matrix accumulation with the aim of targeting the homeostatic state of IVD-matrix metabolism by modulating the factors involved in synthesis and degradation towards the most favourable state to ensure their metabolic balance. Only recently, considerable attention has been placed on the use of stem cells as a clinically applicable therapeutic option for IVD degeneration. 

The aim of this paper was to report the state of the art on the treatment of IVD degeneration (IVD-d), with mesenchymal stem cells (MSCs) based on the *in vitro* and *in vivo* experiences currently available.

## 2. IVD Degeneration 

An active regulation and balance between anabolism and catabolism of the IVD cells is responsible for the maintenance of the physiological homeostasis in IVD tissue.

This mechanism is guaranteed through a complex and accurate coordination of the effect of a number of substances and molecules, including growth factors, enzymes, enzyme inhibitors, and cytokines, that act in a paracrine or/and autocrine way.

IVD degeneration commonly begins during the second decade of life, and it progresses towards severe conditions with ageing [17].

The lack of an adequate nutrient supply [8], along with the inappropriate mechanical load [20], may cause loss and alteration, as well as dysfunction, of cell viability and IVD properties. 

IVD herniation, radiculopathy, myelopathy, spinal stenosis, instability, and low back pain represent the common medical conditions usually associated with symptomatic IVD-d, and they represent the most common among the conditions managed by spine surgeons, in addition to being the leading cause of disability in people younger than 45 years of age.

Various therapeutic strategies have been proposed for IVD-d in the last decade, with the most frequently used ones consisting of surgical interventions characterized by discectomy with vertebral fusion or, as an alternative, conservative management of IVD-d (e.g., lifestyle changes, physical therapy, pain medication, rehabilitation). Neither of such approaches deals with the loss of IVD tissue, and therefore new biological strategies appear challenging.

## 3. MSCs in IVD Degeneration (IVD-d)

Researchers and clinicians have focused their attention on tissue engineering and regenerative medicine to identify long-term tissue repairing options offering the possibility to restore IVD tissues [21].

Attention has been posed on stem cells as a potential source of cells to repopulate and regenerate the IVD. There is a large number of potential sources of MSCs, including adipose tissue, bone marrow, and other tissues [22, 23] as embryonic and fetal stem cells, which are pluripotent cells with a potential to differentiate into any body tissue. Regarding the embryonic stem cells, these cells are highly flexible since they are able to differentiate into any type of tissue, but both the procurement of human embryonic stem cells and research are limited, given the related ethical issues [24].

In terms of IVD regeneration, many studies have shown that IVD-d can be treated or repaired with the injection of MSCs, multipotent cells able to differentiate into tissues of mesenchymal origin including bone, cartilage, fat, muscle, and fibrous tissues depending on the biological environment. These cells can be obtained from multiple adult tissues, including bone marrow, trabecular bone, articular cartilage, muscle, and adipose tissue, which represent a variant of the adult stem cell.

Several authors have speculated that MSCs can also differentiate into the chondrocyte-like cells present in the NP, but, since the phenotype of NP cells has not been clearly characterized yet, confirmation of the differentiation from MSCs to NP cells has not been achieved [23, 25, 26], mainly because AF, NP, and EPs components have a different cartilage or bone composition and different kinds of matrix macromolecules and proteins.

While a variety of cell sources in the field of stem cell research have been proposed for clinical application [21], autologous adipose stem cells (ASCs) as a cell source in bone and cartilage repair have gained significant attention. In fact, adipose tissue is considered a suitable source of stem cells for clinical use, given the easiness of the procedure to retrieve adipose tissue, since this procedure is minimally invasive and can be performed in outpatient clinics and also due to the larger number of cells obtained [27]. Treatment or repair of IVD can occur with an injection of autologous adipose stem cells (ASCs), in presence or absence of growth factor prestimulation or matrix attachment [28].

## 4. Mesenchymal Stem Cells (MSCs) 

### 4.1. *In Vitro* Evidences on MSCs

Based on the high incidence of symptomatic IVD-d caused by disc herniation, radiculopathy, myelopathy, spinal stenosis, instability, and low back pain, there is a great interest around the use of MSCs to repair degenerated IVD tissues. Since the IVD phenotype of NP cells has not been clearly defined yet, there is still a lack of knowledge about differentiation from MSCs to NP cells, complicated by the fact that AF, NP, and EPs components are differently composed of cartilage or bone and matrix. 

Nevertheless, *in vitro* experiments have demonstrated that the application of growth factors and specific culture conditions can guide MSCs to differentiate into particular cell phenotypes [29].

In an *in vitro* study conducted with MSCs cultured in the presence of TGF-b, it was observed that the MSCs were able to express a phenotype similar to NP cells with the production of type II collagen and aggrecan [30]. Moreover, under specific culture conditions such as hypoxia, it was initially seen that hypoxia contributed to maintain an NP cell phenotype in *in vitro* cultures, and subsequently the combination of hypoxia and TGF-b1 exposure proved to advance differentiation of the MSCs into an NP-like phenotype. On the basis of this experience, researchers suggested that the hypoxic environment of the IVD could promote differentiation of the MSCs towards an NP-like phenotype *in vivo* [31, 32].

The effect of cocultures of MSCs with AF and NP cells obtained from human degenerative IVDs in 3-dimensional pellet cultures has also been investigated. The coculture determined an increased proliferation in both the MSC-AF and MSC-NP cultures, but a higher proteoglycan production was found only in the MSC-AF coculture. Based on the interaction between AF cells and MSCs, a direct implantation of the MSCs *in vivo* may be applied without any need to differentiate the MSCs before implantation [33]. 

In their research in the IVD implantation of MSCs, Sakai et al. [34–36] initially implanted MSCs into a normal IVD and subsequently repeated the same experiment in a IVD degeneration model in rabbit. Based on the results obtained in their initial study, they reported that autologous MSCs implanted into a rabbit IVD survive and proliferate, with differentiation into a NP-like phenotype. At 48 weeks since implantation, labelled MSCs (i.e., tagged with the gene for green fluorescent protein (GFP) with the GFP-labelled cells followed tracking their effects for a period of 48 weeks) expressed factor-a (HIF-a), glutamine transporter-1, and MMP-2, which represent hypoxia-inducible NP phenotypic-related markers [36]. Autologous MSCs injected into the IVD could reestablish a chondrocyte-like cell population able to produce proteoglycans and collagen II in a rabbit degenerating IVD. At MRI imaging, degenerating IVDs treated with MSCs showed improvement compared to controls both in IVD height and hydration [35]. 

To repair the matrix of degenerative NP tissue, transplanted cells must produce large quantities of collagen, proteoglycans (e.g., aggrecan), and other matrix proteins [37]. Generally, these matrix proteins are produced by cells located in the central regions of the IVD and chondrocytes, making them candidates for cell-based IVD repair. Also, progress recorded in stem cell research in the past decade provides an attractive scenario for the use of adult MSCs (ASCs) for NP tissue engineering [30, 38–46]. Thereafter, while a variety of cell sources in the field of stem cell research has been proposed for clinical application [21], ASCs as a cell source in bone and cartilage repair have gained significant attention. Particularly, application of ASCs in NP tissue regeneration has been the subject of a number of recent studies [27, 47–49]. One study reported that the differentiation of ASCs into a NP cell-like phenotype occurred in coculture of human ASCs and NP cells in a micro-mass-cultured mode. In other experiments, expression of proteoglycan and type I collagen in 3D-cultured sand rat ASCs could be significantly stimulated either through treatment of TGF-b or coculture of human IVD cells [27, 49]. 

Moreover, in coculture of NP cells and ASCs combined in micromasses, separated by a permeable membrane to allow the diffusion of soluble factors, an upregulation of the aggrecan and collagen II expression was detected [50]. Therefore, soluble factors released by NP cells may have a direct effect on promoting the differentiation of the ASC into the NP-type lineage [51]. 

Coculture of human NP and ASCs improves the quality of the tissue reconstructed *in vitro* both in terms of 3D cell organization and matrix production [47]. Conversely, cell expansion is essentially needed to obtain sufficient cell quantities for transplantation, and the common monolayer culture approach has determined a number of concerns including cell dedifferentiation, senescence, and genetic mutagenesis [40]. 

However, adipose tissue might be considered as an adequate source of stem cells for clinical use, based both on the ease of the procedure to retrieve adipose tissue and the cell quantities that can be obtained. In fact, on one hand, the collection of adipose tissue occurs through a minimally invasive procedure allowing for this to be straightforwardly performed in outpatient clinics, and, on the other hand, it allows for yields of adherent ASCs to reach up to 25,000/g of tissue. Therefore, although somewhat new in the stem cell research area, ASCs have gained intensive attention as a cell source in bone and cartilage repair [27] and the use of ASCs as seed cells in NP tissue engineering has been the subject of further investigations [52, 53]. 

The results of cocultured rabbit ASCs with NP tissues through a specifically designed device showed that ASCs responded to soluble mediators from NP tissues [53]. In experimental studies, NP or AF tissues were cocultured with alginate beads containing ASCs isolated from inguinal fad pads of NZW rabbits and following real-time RT-PCR analysis and it was observed that NP tissues could notably stimulate type II collagen and aggrecan genes expression in ASCs, whereas this was not the case for AF tissues [53]. 

Recently, confirmation of the feasibility of a stem cell therapy for IVD repair has been obtained by a parallel *in vitro* and *in vivo* study. In the *in vitro *portion of the study, in which bone-marrow-derived stem cells (BSCs) and IVD cells were cocultured, the results showed an improvement of ECM production, while the *in vivo* study persistence of BSC for at least 24 weeks after implantation in rabbit IVD was shown [54].

### 4.2. *In Vivo* Evidences on MSCs

The future of treatment options for IVD degeneration should deal with evidences regarding the use of MSCs in animal studies. 

The possibility of allogeneic MSC transplantation has also been suggested and should be considered [55], based on a number of potential advantages of allograft stem cells versus autograft stem cells.

It is theorized that the use of autograft MSCs may have the same genetic predisposition for degeneration as the native NP cells, while it should be possible to escape this genetic predisposition by using an allograft cell population. The use of allograft MSCs may in fact allow for the selection and maintenance of a cell population that is less likely to undergo degeneration. Also, in comparison to collection and culture of MSCs for each individual patient, allograft cells could be maintained for “off the shelf use” with a higher convenience and reduced costs [24].

Several *in vivo* animal experiments conducted with the implant of MSCs into the IVD have highlighted promising results in terms of long-term viability of the implanted cells and differentiation into a phenotype similar to the native NP cells with the production of type II collagen and aggrecan [24].

Crevensten et al. [56] were the first in 2004 to report the viability of allograft MSCs implanted in a rat IVD. They observed the proliferation of the stem cells as well as a trend towards increased IVD height after a 4-week follow-up period, which suggested an enhanced production of proteoglycans. 

Subsequently, allograft MSCs were also transplanted into the New Zealand white rabbit: the cells survived, proliferated, and differentiated into native NP cells like phenotype. After grafting, the animals were followed for 6 months, and no immune reactions occurred towards these allograft cells. In the IVDs treated with MSCs, type II collagen production along with an increased proteoglycan concentration was detected [57].

The effectiveness of MSC transplantation has also been confirmed in large animal models (chondrodystrophoid breed canine with nucleotomy) closer to humans. In fact, in a canine model of IVD degeneration, MSC transplantation determined a positive outcome on preservation of immune privilege, possibly by differentiation of the transplanted MSCs into FasL expressing cells [38]. These data suggest that the NP region may tolerate MSCs from other areas of the body or even other individuals [58].

Other researchers have also investigated the use of allogenic MSCs for IVD regeneration, despite the concerns for immunologic rejection of the allograft tissue. Similarly to previous experiences showing *in vivo* viability of allograft NP tissue, the “immunologically privileged” environment of the avascular IVD space may also allow for long-term viability of allograft stem cells [59, 60].

The ability of allogenic MSCs to escape alloantigenic immune recognition and determine an immunomodulatory effect has been noted also in other studies [61, 62]. The ability to elude immune recognition may arise from the lack of major histocompatibility complex class II molecules on MSCs. Nevertheless, major histocompatibility complex class II expression has been observed as MSCs differentiate or are exposed to inflammatory cytokines [61, 62]. 

An immunomodulatory effect of MSCs on the local environment by producing anti-inflammatory cytokines such as TGF-b and IL-10 has also been reported [61, 63]. In addition to enhancing the probability for MSC graft survival, this immunomodulatory effect of MSCs may also play a role in IVD regeneration by modifying the environment from a proinflammatory, catabolic condition to a more anti-inflammatory, anabolic environment [4–6, 24, 64, 65].

Several studies have now confirmed the effectiveness of MSC transplantation, demonstrating that MSCs transplanted into degenerating IVDs* in vivo* can survive, proliferate, and differentiate into cells expressing the phenotype of NP cells with suppression of inflammatory genes [35].

There are a large number of different types of stem cells and a variety of possible sources of MSCs including bone marrow, adipose tissue, and other tissues that have received significant attention as potential sources of cells to repopulate and regenerate the IVD.

Bone marrow aspiration, which has been used by spinal surgeons in fusion procedures, is an excellent source of MSCs [24]. When bone-marrow-derived stem cells (BSCs) combined with a hyaluronic acid-derived scaffold were injected into pig IVD, IVDs presented a central NP-like region. This finding suggested that BSC are able to differentiate in IVD-similar cells and also to produce matrix proteins similar to normal IVD tissues [66]. Further confirmation comes from experiments performed in previously degenerated or degraded IVDs in rabbits, that showed how, following transplantation in these IVDs, BSCs were able to proliferate and differentiate into cells expressing and producing some of the main, although not all, extracellular components of the IVD [67]. 

Recently, other stem cells such as synovial MSCs have been studied as a potential source of cells to be used for IVD-d. In an induced IVD-d model in rabbits, allogenic synovial MSCs have been transplanted and evaluated up to 24 weeks postoperatively with imaging analyses such as magnetic resonance imaging, radiographs, and histological analysis. T2-weighted MR imaging revealed a higher signal intensity of the NP in the MSC group. Radiographs showed that the height of IVD in the MSC group remained higher compared to that in the degeneration group. Immunohistochemical analyses showed a higher expression of type II collagen around NP cells in the MSC group compared to that of the normal group. These results brought to the conclusion that synovial MSCs injected into the NP space promote synthesis of the remaining NP cells to type II collagen as well as inhibition of degrading enzymes and inflammatory cytokines expression, resulting in the structure of the intervertebral IVD being maintained [68].

Several authors have recently reported their successful results using different types of stem cells, but there is still a lack of published evidence regarding adipose-derived stem cells (ASCs) implantation for IVD degeneration management, and, only recently, experiments with ASC have confirmed this type of stem cells as another potential cell source for IVD repair and regeneration. In fact, in a goat ABC chondroitinase degeneration model [69], ASC can be beneficial for cell therapy of IVD disease because these cells are easily isolated compared to other MSCs such as BSCs [70, 71].

Despite the promising results deriving from animal studies, there is still a lack of studies performed in humans. 

To our knowledge, the only trial conducted in humans, a nonrandomized, noncontrolled study evaluating autologous hematopoietic stem cells transplanted into IVD for the treatment of low back pain in 10 patients, did not report any significant improvement of the patients' condition [72]. 

Therefore, further research and randomized controlled studies are required for a better definition of the effectiveness of the administration of ASC, rather than BSC or other types of stem cells for the management of IVD degeneration.
